# Work at inpatient care units is associated with an increased risk of SARS-CoV-2 infection; a cross-sectional study of 8679 healthcare workers in Sweden

**DOI:** 10.1080/03009734.2020.1793039

**Published:** 2020-07-20

**Authors:** Anna-Karin Lidström, Fredrik Sund, Bo Albinsson, Johan Lindbäck, Gabriel Westman

**Affiliations:** a Department of Medical Sciences, Section of Infectious Diseases, Uppsala University, Uppsala, Sweden; b Laboratory of Clinical Microbiology, Uppsala University Hospital, Uppsala, Sweden; c Uppsala Clinical Research Center, Uppsala University, Uppsala, Sweden

**Keywords:** Covid-19, healthcare workers, IgG, SARS-CoV-2, transmission

## Abstract

**Background:**

During the Covid-19 pandemic, the protection of healthcare workers has been in focus throughout the world, but the availability and quality of personal protective equipment has at times and in some settings been suboptimal.

**Materials and methods:**

A total of 8679 healthcare workers and healthcare support staff in the county of Uppsala, north of Stockholm, were included in this cross-sectional study. All subjects were analysed for IgG anti-SARS-CoV-2, and predictors for positive serostatus were analysed in a logistic regression model including demographic parameters and self-reported employment characteristics.

**Results:**

Overall, 577 (6.6%) were classified as seropositive, with no statistically significant differences between healthcare workers and support staff. Among healthcare workers, age (OR 0.987 per year, 95% CI 0.980–0.995), time to sampling (OR 1.019 per day, 95% CI 1.004–1.035), and employment at an outpatient care unit (OR 0.620, 95% CI 0.487–0.788) were statistically significantly associated with risk of infection. Covid-19 specific units were not at particular risk, compared to other units with comparable characteristics and staff demography.

**Conclusion:**

Our findings indicate that SARS-CoV-2 transmission is related to inpatient healthcare work, and illustrate the need for a high standard of basic hygiene routines in all inpatient care settings.

## Introduction

The SARS-CoV-2 pandemic began in late 2019, originating from the Hubei province in China (1). Compared to seasonal influenza, the pattern of human-to-human transmission appears more clustered around super-spreaders of the virus, causing national and regional differences that are yet to be completely understood (2).

Similar to the SARS-CoV-1 outbreak in 2003–2004, there were early reports of frequent transmission to healthcare professionals, including several with fatal outcome (3,4). Even though most countries put extensive effort into acquiring personal protective equipment in order to provide adequate protection for caregivers, numerous reports describe shortages in quantity or quality of the protective equipment provided (5). In Swedish hospital units dedicated to care for Covid-19 patients, single-use protective masks were by necessity to a large extent replaced by multi-use filter masks intended for military or civilian purposes. Although the technical filter performance is superior to FFP3-class devices, the total protective effect of these devices in a healthcare setting is not known (6).

The adaptive immune response to SARS-CoV-2 infection is multi-faceted, including both cellular and humoral components, but for diagnostic purposes the development of virus specific IgG has been a gold standard for convalescent patients where RNA no longer can be amplified from upper airway specimens (7). Early in the pandemic point-of-care antibody tests dominated the market but have now to a variable extent been replaced with antibody detection kits on high throughput platforms. The sensitivity and specificity of the Abbott Architect SARS-CoV-2 IgG assay has previously been evaluated, but it is still not clear to what extent the severity of Covid-19 disease affects the level of seroconversion. This could result in lower sensitivity in populations with mild or asymptomatic disease (8).

Region Uppsala is a Swedish public health region located just north of Stockholm, delivering health care to the population in the county of Uppsala as well as specialized care in the university hospital to inhabitants referred from surrounding counties. From 27 May 2020, all healthcare and healthcare administrative support staff in Region Uppsala were offered testing for IgG anti-SARS-CoV-2. Here, we present the rate of infection and investigate professional and demographic factors associated with transmission.

## Materials and methods

### Study subjects

Between 27 May and 25 June 2020, all healthcare staff, including support staff, in Region Uppsala were offered free testing for IgG anti-SARS-CoV-2 within the study. The largest employers include Uppsala university hospital (approximately 8000 employees), primary health care (approximately 1400 employees), and Enköping hospital (approximately 550 employees). However, as also part-time workers and private healthcare providers were allowed to participate, the exact number of potential participants is unknown. Study subjects were to be above the age of 18 and without symptoms of airway infection for at least seven days. After informed consent was obtained, sampling was performed in line with clinical routines at their unit of employment or at general sampling units, at the discretion of the study subject.

All subjects’ self-reported place of work was coded in the analysis into three independent variables: primary versus hospital care, outpatient versus inpatient care, and Covid-19 (specific and possible) units versus other units. Coding was done up to the level of detail provided by the study subject, with missing data leading to differences in the denominators presented in the results section. In Covid-19 possible units such patients could have been cared for, whereas in Covid-19 specific units most patients were diagnosed with Covid-19. Personal protective measures in Covid-19 possible and specific units (including intensive care units) were standardized and included both multi-use filter face masks and long-sleeved gowns at all times.

The study was approved by the Swedish Ethical Review Authority (no. 2020–02688).

### IgG anti-SARS-CoV-2 immunoassay

The analysis of SARS-CoV-2 IgG antibodies was carried out using the CE-labelled SARS-COV-2 IgG kit with nucleoprotein-based antigen on the Architect i2000SR Analyser (Abbott, Abbott Park, IL, USA).

The assay is an automated, two-step immunoassay for detection of IgG antibodies to SARS-CoV-2 in human serum and plasma using chemiluminescent microparticle immunoassay (CMIA) technology.

Sample, SARS-CoV-2 antigen-coated paramagnetic microparticles, and assay diluent are combined. The IgG antibodies to SARS-CoV-2 present in the sample bind to SARS-CoV-2 antigen-coated microparticles. After washing, an anti-human IgG acridinium-labelled conjugate is added to create a reaction mixture. Following a wash cycle, pre-trigger and trigger solutions are added. The resulting chemiluminescent reaction is measured as a relative light unit (RLU). The presence or absence of IgG antibodies to SARS-CoV-2 in the sample is determined by comparing the chemiluminescent RLU in the reaction to the calibrator RLU. There is a direct relationship between the amount of IgG antibodies to SARS-CoV-2 in the sample and the RLU detected by the system optics. This relationship is reflected in the calculated index (S/C). A positive/negative cut-off of 1.4 S/C was used in line with the manufacturer’s instructions.

### Statistical analyses

Statistical analyses were performed with R version 3.4.1, with packages plyr version 1.8.4 and ggplot2 version 3.0 (9). Proportions of seropositive subjects were estimated and compared using uni- and multivariable logistic regression models. The association with age and calendar time was assumed to be linear on the log-odds scale. Unadjusted results are presented as the estimated prevalence with corresponding 95% confidence intervals. Associations between the prevalence and the studied factors in the multivariable models are presented as odds ratios with corresponding 95% confidence intervals and *p* value less than 0.05. For all tests, a statement of statistical significance implies a *p* values less than 0.05.

## Results

### IgG anti-SARS-CoV-2 immunoassay

**Figure 1.**
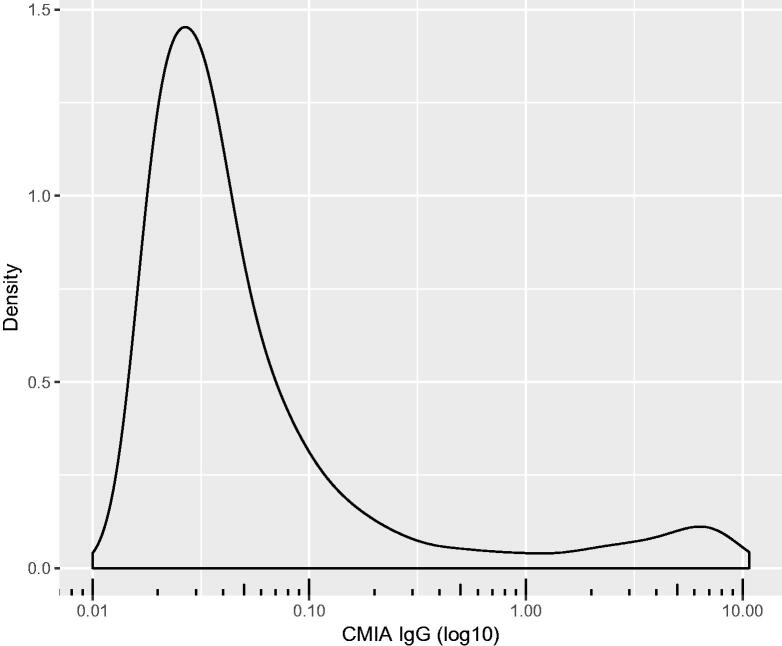
Density plot illustrating the distribution of IgG anti-SARS-CoV-2. All values shifted 0.01 to allow logarithmic transformation. All subjects included in the analysis.

During the four-week testing period a total of 8679 individuals (77% women and 23% men) participated in the study. All blood samples were analysed for IgG anti-SARS-CoV-2, with 577 (6.6%) positive results using the manufacturer’s cut-off at 1.4 S/C. Antibody levels were distributed into two populations with a geometrically neutral cut-off at approximately 1.0 S/C (Figure 1). This is in line with a borderline result category recently introduced in clinical use between 0.9 and 1.39 S/C, which would expand the seropositive population to 635 (7.3%) subjects.

### Baseline characteristics and subgroup prevalence

**Figure 2.**
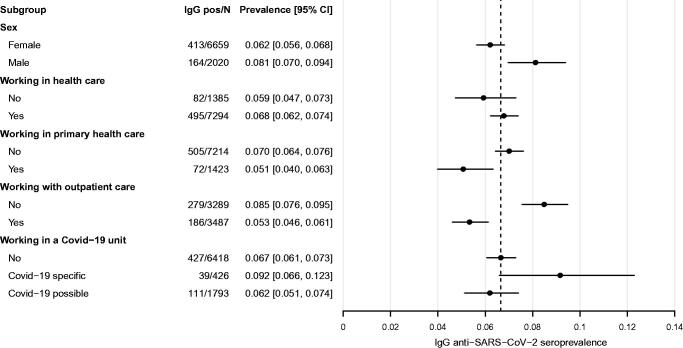
Forest plot of IgG anti-SARS-CoV-2 prevalence. Subgroup prevalence and confidence intervals of IgG anti-SARS-CoV-2 positivity. N is the total number of subjects in each category. All subjects included in the analysis.

IgG positive study subjects were slightly younger and more often working at an inpatient care unit, compared to IgG negative subjects. Evaluation of the unadjusted subgroup prevalence of IgG anti-SARS-CoV-2 seropositive subjects shows higher proportions of IgG positive subjects among male participants and those working in inpatient care units and Covid-19 specific units (Table 1; Figure 2).

**Table 1.** Characteristics of IgG anti-SARS-CoV-2 positive and negative study subjects.

	IgG positive	IgG negative
Age, years	42 (18–78)	45 (18–85)
Sampling time from project start, days	12 (0–28)	12 (0–29)
Male sex	164/577 (28.4%)	1855/8102 (22.9%)
Working in health care	495/577 (85.8%)	6799/8102 (83.9%)
Working in primary health care	72/577 (12.5%)	1351/8060 (16.8%)
Working with outpatient care	186/465 (40.0%)	3301/6311 (52.3%)
Working in Covid-19 specific unit	39/577 (6.8%)	387/8060 (4.8%)
Working in Covid-19 possible unit	111/577 (19.2%)	1682/8060 (20.9%)

Data presented as medians (range) or proportions.

### Predictors of IgG anti-SARS-CoV-2 serostatus

To analyse the overall effect of healthcare employment versus administrative support employment on the risk of SARS-CoV-2 infection, a logistic regression model was created using age, gender, and sampling time as covariates (Table 2). Working with direct patient contact versus healthcare support did not appear to have a statistically significant effect, but lower age and male sex were both associated with an increased risk of infection.

**Table 2.** Predictors of IgG anti-SARS-CoV-2 serostatus.

	Odds ratio	95% confidence interval	*p* Value
Age, years	0.984	0.978–0.991	<0.001
Time from study start, days	1.005	0.992–1.019	0.41
Male sex	1.334	1.104–1.612	0.003
Working in health care	1.175	0.918–1.505	0.2

Multivariable logistic regression model. All study subjects included in the analysis.

**Figure 3.**
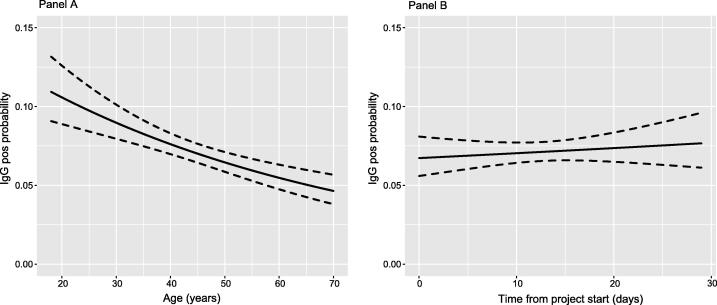
Univariable logistic modelling of relation between age (Panel A, left) and time to sampling (Panel B, right) and risk of IgG anti-SARS-CoV-2 positivity. All subjects included in analysis.

Subsequently, the risk of SARS-CoV-2 infection in relation to type of workplace was analysed in a logistic regression model using the same covariates as described above but including only staff with direct healthcare tasks. In addition to lower age and later sampling time (Figure 3), also work at an inpatient care unit was statistically significantly associated with an increased risk of infection (Table 3). There were no statistically significant risk differences related to working at Covid-19 specific care units or at primary healthcare centres.

**Table 3.** Predictors of IgG anti-SARS-CoV-2 serostatus.

	Odds ratio	95% confidence interval	*p* Value
Age at sampling, years	0.988	0.980–0.995	0.001
Time from study start, days	1.019	1.004–1.035	0.014
Male sex	1.107	0.879–1.394	0.387
Working in primary health care	0.711	0.493–1.026	0.068
Working with outpatient care	0.631	0.497–0.801	<0.001
Working in Covid-19 specific unit	1.114	0.766–1.619	0.572
Working in Covid-19 possible unit	1.275	0.945–1.721	0.112

Multivariable logistic regression model. Only healthcare staff included in the analysis.

## Discussion

The Covid-19 pandemic has put healthcare systems across the world at stress, to an extent that has rarely been seen in developed countries. In Sweden, the just-in-time logistics system for delivering personal protective equipment failed within days to weeks when the demand quickly increased to a level that was beyond the imagination of the regional pandemic planning in early 2020 (10).

Given that staff availability and experience are key to healthcare capacity and quality, protecting healthcare professionals from hospital-acquired infections is pivotal. In contrast to early reports of numerous cases of severe infections and deaths among Chinese and Italian healthcare professionals (3,4), the overall rate of IgG anti-SARS-CoV-2 in our study is relatively low. However, our analyses show that healthcare workers at inpatient care units have a statistically significantly increased risk of infection that cannot be explained by demographic differences or transmission at Covid-19 specific care units only. The age-dependent association likely represents two factors, both a higher level of community transmission among the young, and a correlation between age and level of physical contact with patients during inpatient care, which relates to differences in length of training and the pyramid-shaped age-related hierarchy among different categories of clinical staff.

Our findings emphasize the need for a high standard in basic hygiene routines in all settings, especially in inpatient care where physical contact with patients is more extensive, as Covid-19 patients are not always easily identifiable through symptom-based triage. In contrast, Covid-19 specific units with routines of high hygiene standard and protective measures do not appear to be at an additional risk compared to other comparable inpatient and outpatient care settings.

The performance of the IgG anti-SARS-CoV-2 immunoassay used in this study is well characterized in patients with symptomatic disease, but less is known about PCR-positive patients with mild or asymptomatic disease where the rate of IgG seroconversion has been shown to be lower (11). As this affects the interpretation of seroepidemiological data, studies are needed to further characterize the cellular immune response and cross-reactivity with other circulating corona viruses (12). However, this should not affect our within-study comparisons of healthcare worker sub-groups.

We believe our results are relevant and generalizable to other Swedish healthcare settings and to some extent also to international inpatient care settings. Also, our findings are comparable with previously published studies of seroprevalence in healthcare workers in contact with Covid-19 patients (13). The total rate of IgG positivity and the temporal trend indicate ongoing community transmission of SARS-CoV-2 in the Uppsala County. However, the vast majority of the population has not been infected. This is in line with the current knowledge of the cluster-like spread of the virus and has implications for public health strategies. Unless there is a high proportion of occult infection not resulting in IgG seroconversion detected by the assay used in this study, the remaining spread of SARS-CoV-2 needed to achieve herd immunity appears to be a goal too far away to be feasible in terms of the associated disease burden.
